# Supplementary figures and images for: Effects of Below-Ground Microbial Biostimulant Trichoderma harzianum on Diseases, Insect Community, and Plant Performance in Cucurbita pepo L. under Open Field Conditions
Source: Microorganisms. 2022 Nov 12;10(11):2242. doi: 10.3390/microorganisms10112242 (PMC9692614; doi:10.3390/microorganisms10112242)

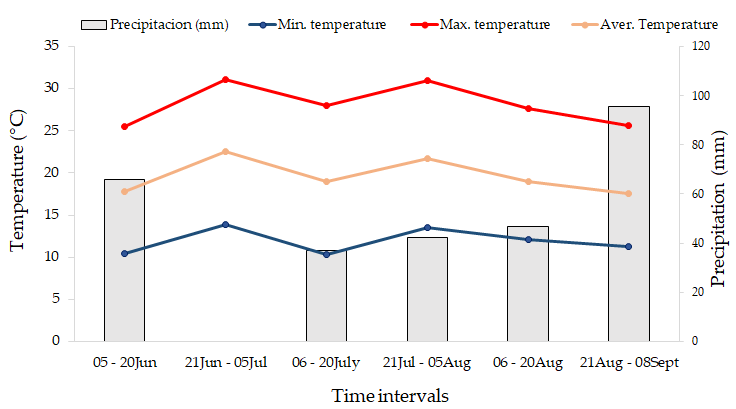

Supplement: Supplementary file 1 [file microorganisms-10-02242-s001.zip › Figure S1.tif]

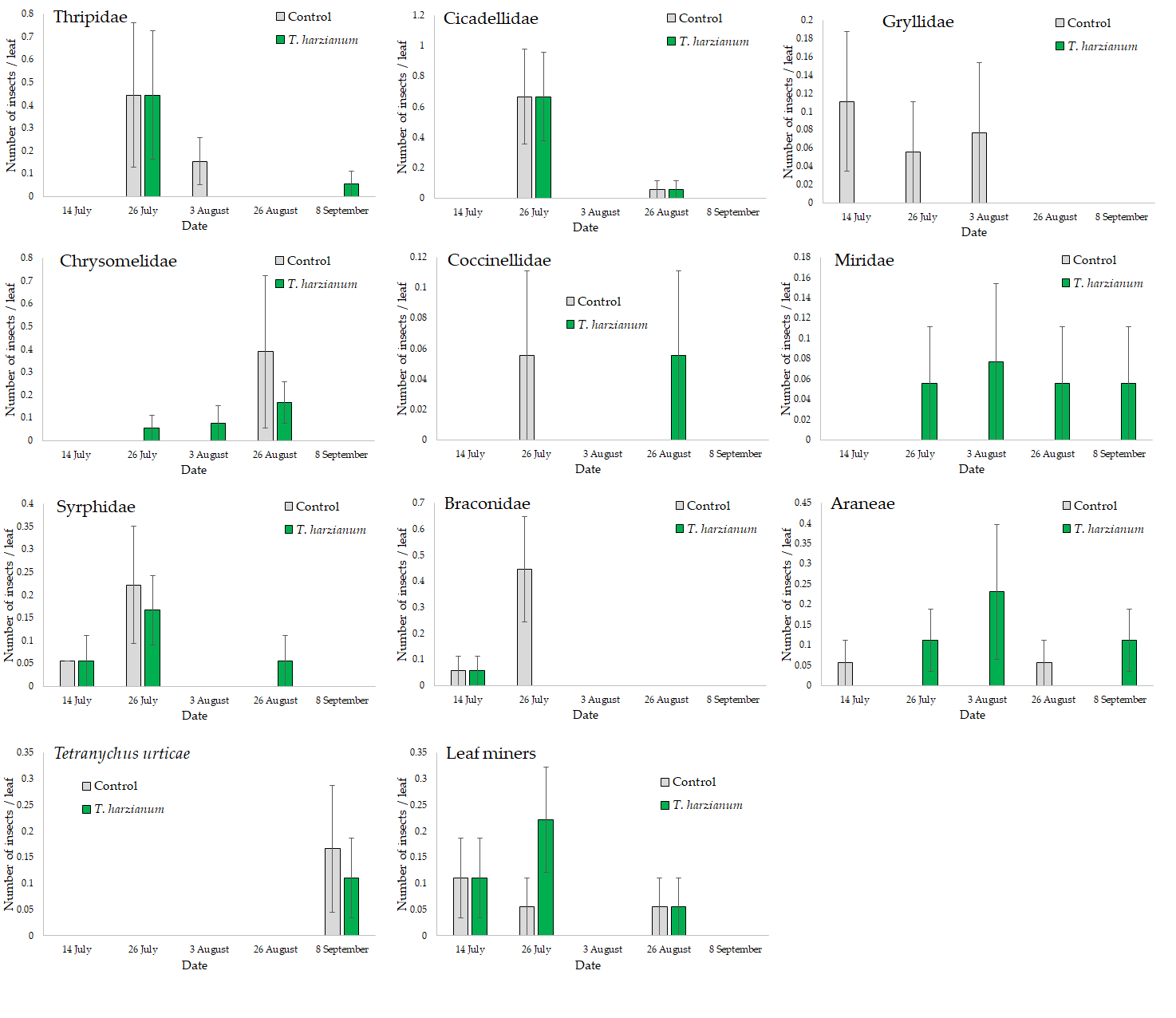

Supplement: Supplementary file 1 [file microorganisms-10-02242-s001.zip › Figure S2.tif]

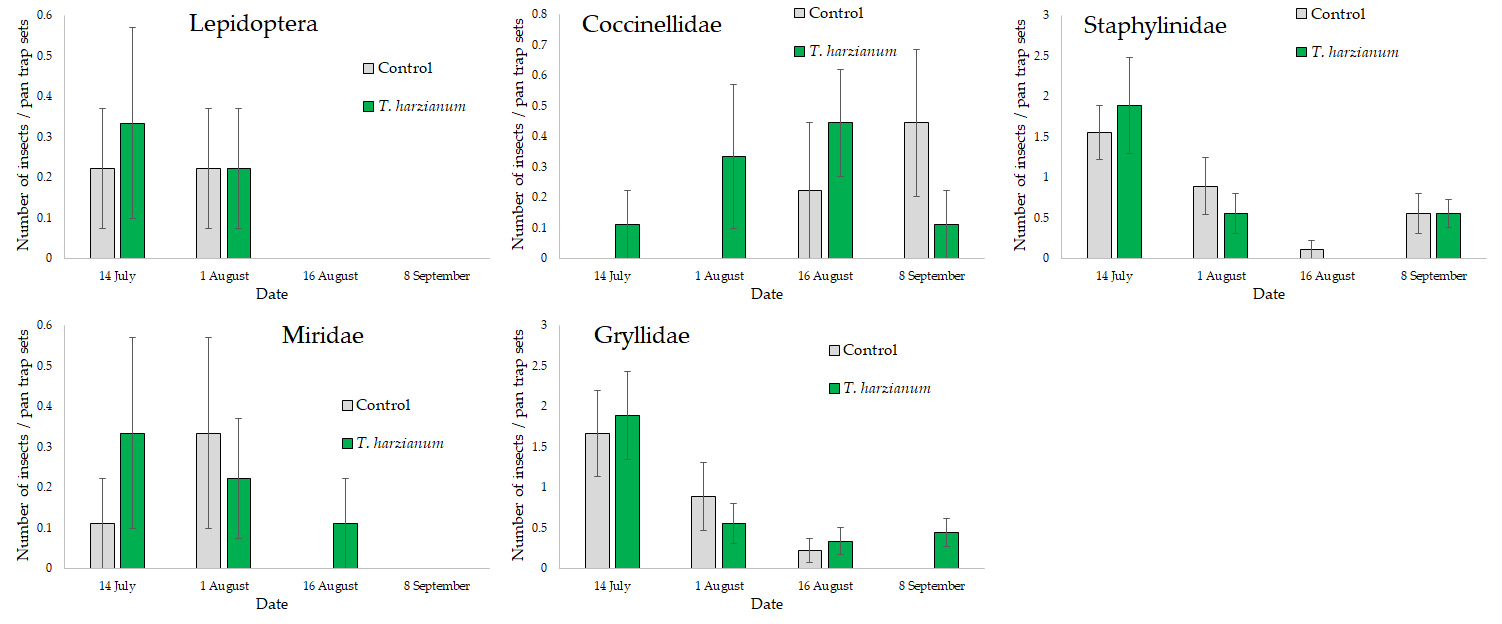

Supplement: Supplementary file 1 [file microorganisms-10-02242-s001.zip › Figure S3.tif]
